# Simultaneous correction of leg length discrepancy and angular deformity of the distal femur with retrograde Precice nails: a retrospective analysis of 45 patients

**DOI:** 10.2340/17453674.2024.40947

**Published:** 2024-07-15

**Authors:** Bjoern VOGT, Caja BIERMANN, Georg GOSHEGER, Andrea LAUFER, Anna RACHBAUER, Carina ANTFANG, Milena LUECKINGSMEIER, Gregor TOPOROWSKI, Henning TRETOW, Robert ROEDL, Adrien FROMMER

**Affiliations:** 1 Pediatric Orthopedics, Deformity Reconstruction and Foot Surgery, Muenster University Hospital; 2 General Orthopedics and Tumor Orthopedics, Muenster University Hospital, Germany

## Abstract

**Background and purpose:**

Magnetically controlled motorized intramedullary lengthening nails (ILNs) can be employed for simultaneous correction of angular deformities of the distal femur and leg length discrepancy. This spares typical complications of external fixators but requires precise preoperative planning and exact intraoperative execution. To date, its results are insufficiently reported. We aimed to elucidate the following questions: (i) Is acute angular deformity correction and gradual femoral lengthening via a retrograde ILN a reliable and precise treatment option? (ii) What are the most common complications of treatment?

**Methods:**

Acute angular deformity correction and subsequent gradual lengthening of the distal femur with retrograde ILN was retrospectively analyzed in 45 patients (median patient age: 15 years, interquartile range [IQR] 13–19 and median follow-up: 40 months, IQR 31–50). Outcome parameters were accuracy, precision, reliability, bone healing, and complications of treatment.

**Results:**

The median distraction was 46 mm (IQR 29–49), median distraction and consolidation index 0.9 mm/day (IQR 0.7–1.0) and 29 days/cm (IQR 24–43), respectively. The median preoperative mechanical axis deviation (MAD) was 30 mm (IQR 23–39) in the varus cohort and –25 mm (IQR –29 to –15) in the valgus cohort and reduced to a mean of 8 mm (standard deviation [SD] 8) and –3 (SD 10), respectively. Accuracy, precision, and reliability of lengthening were 94%, 95% and 96%, respectively. Accuracy and precision of deformity correction were 92% and 89%, respectively. In total, 40/45 of patients achieved distraction with a difference of less than 1 cm from the initial plan and a postoperative MAD ranging from –10 mm to +15 mm. In 13/45 patients unplanned additional surgeries were conducted to achieve treatment goal with nonunion being the most frequent (4/45) and knee subluxation (3/45) the most severe complication.

**Conclusion:**

Acute deformity correction and subsequent lengthening of the distal femur with retrograde ILN is a reliable and accurate treatment achieving treatment goal in 89% but unplanned additional surgeries in 29% of patients should be anticipated.

Distraction osteogenesis with intramedullary lengthening nails (ILNs) is an established alternative to external fixators and has shown reliable and accurate results, sparing typical complications of external fixation [1-5]. Regarding femoral ILNs, an antegrade nail insertion is suitable for straight gradual lengthening without concomitant correction of additional distal femoral deformities [1,2,6], whereas a retrograde nail implantation enables simultaneous acute 3-dimensional deformity correction and subsequent gradual lengthening at the distal femur [7,8]. Alternatively staged treatment with distal femoral hemi-epiphysiodesis or distal femoral correction osteotomy with plate fixation and subsequent femoral lengthening via an antegrade ILN can be conducted. [9,10]. While surgical techniques of retrograde femoral nail insertion and application of blocking screws for acute angular deformity correction are well described [7,11], to date, the outcome of femoral lengthening with retrograde ILNs for correction of leg length discrepancy (LLD) and angular deformity of the distal femur is insufficiently reported. Present studies are heterogeneous in terms of the applied implants and operative approaches for nail insertions, and provide relatively short follow-up periods [4,5].

We aimed to elucidate the following questions: (i) Is acute angular deformity correction and gradual femoral lengthening via a retrograde ILN a reliable and precise treatment option? (ii) What are the most common complications of treatment?

## Methods

### Patients and indications

**Figure 1:**
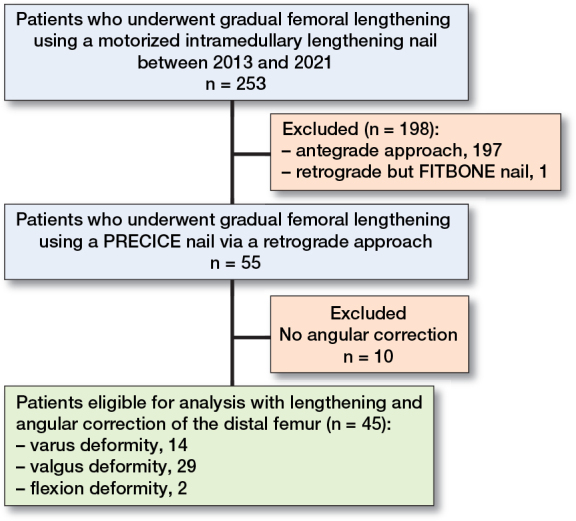
Patient flowchart.

The longitudinally maintained database of our orthopedic teaching hospital was retrospectively analyzed to identify all patients who underwent simultaneous angular deformity correction of the distal femur and distraction osteogenesis for correction of LLD via retrogradely inserted ILNs between 2013 and 2021. A total of 45 patients (19 females, 18 left femora) with a median age of 15 years (interquartile range [IQR] 13–19) and a median follow-up of 40 months (IQR 31–50) were found to be eligible for analysis (Figure 1). Femoral distraction osteogenesis with retrograde ILNs was not considered for patients with persisting deep tissue infection, unaffected growth plate of the distal femur, bone dimensions too small for implants, and LLD < 2 cm. In all patients the retrograde approach was chosen to concomitantly conduct acute correction of a distal femoral deformity (varus: n = 16, valgus: n = 27, flexion: n = 2) (Table 1). Patients who underwent retrograde femoral lengthening with ILNs without concomitant deformity correction (n = 10) were excluded from analysis. Findings are reported according to the Strengthening the Reporting of Observational Studies in Epidemiology guidelines (STROBE) [12].

**Table 1:** Etiology of the study cohort

Etiology	n
Posttraumatic	13
Postinfectious	10
Congenital femoral deficiency	7
Tumor related growth alternation	5
Post Perthes	2
Post SCFE	2
Iatrogenic ^a^	2
Congenital hip dislocation	1
Other	3

SCFE = slipped capital femoral epiphysis.

a Related to previous surgical treatment.

### Preoperative and postoperative clinical and radiographic assessment

**Figure 2:**
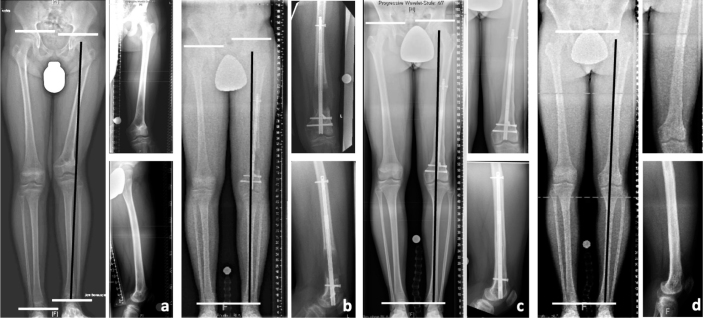
A 13-year-old male patient with left-side post-traumatic femoral shortening (leg length difference (LLD): at operation 38 mm, predicted at skeletal maturity 75 mm) and distal femoral valgus deformity (MAD –23 mm, mLDFA 74°) due to partial closure of the distal femoral growth plate following physeal fracture at the age of 10 years (a). Together with permanent epiphysiodesis of the medial distal femoral growth plate, deformity reconstruction using a retrograde intramedullary lengthening nail (PRECICE, NuVasive) and a blocking bolt for acute angular correction of 12° with subsequent gradual lengthening by distraction osteogenesis was performed (b–d). To take into account the remaining contralateral growth, an intended overcorrection of 37 mm was realized by lengthening by a total of 75 mm (b). The overcorrection decreased continuously showing only mild residual LLD of 10 mm just before implant removal (1.5 years after implantation) at the age of 14.5 years (c). At skeletal maturity at the age of 16 years the patient showed equal leg length (residual LLD 1 mm) and neutral coronal alignment (MAD +1 mm, mLDFA 86°) (d).

**Figure 3:**
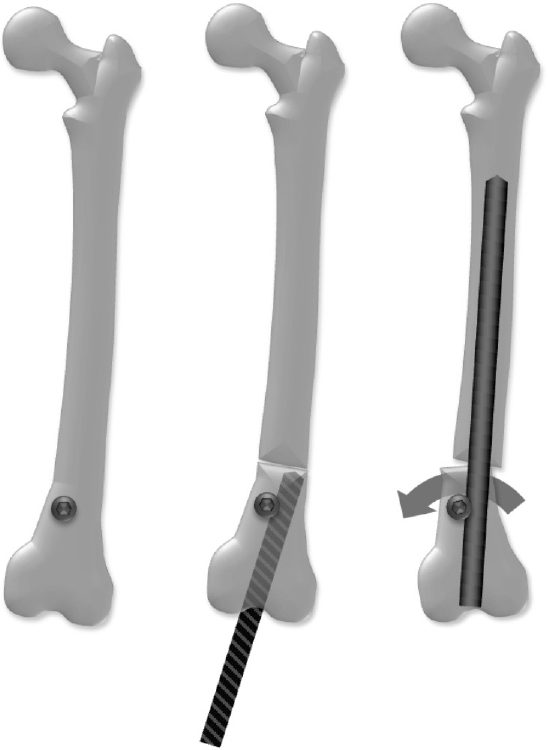
Position of the blocking screws to control the distal femoral fragment by guiding the trajectory of the reamer and nail in the right direction was determined using the following principle: “The blocking screw precedes the direction of fragment rotation for correction.”

Clinical information was acquired from hospital records preoperatively, after distraction, and at maximum follow-up. During distraction, biplanar femoral radiographs were taken every 2 weeks, then every 6 weeks after distraction until consolidation, and analyzed using the Centricity PACS calibrated digital radiology system (GE Healthcare, Chalfont St Giles, UK). Measurements were conducted with the TraumaCAD post-processing software (Brainlab, Munich, Germany). Radiographic analysis also included pre- and postoperative calibrated anteroposterior (AP) long standing radiographs for measurement of mechanical axis deviation (MAD), mechanical lateral distal femoral angle (mLDFA), and LLD, according to established standards [13]. Preoperative planning was conducted according to the reverse planning method [14] (Figure 2). Blocking screws were applied in all patients (Figure 3).

### Surgical technique and perioperative parameters

#### Surgical technique

ILN insertion was conducted with the patient placed in a supine position and a retrograde femoral entry via an infrapatellar either trans- or paraligamentous approach. If there was pre-existing damage to the distal femoral growth plate, a permanent epiphysiodesis of the distal femur was conducted to inhibit unpredictable growth. After arthrotomy of the knee a 3.2 mm tip threaded guide wire was inserted in the intercondylar notch with the help of a honeycomb and an entry portal tube for soft tissue protection (instruments from the Trigen base set, Smith+Nephew, Memphis, TN, USA). After assuring that the guide wire was positioned correctly by biplanar intraoperative fluoroscopy blocking screws were inserted according to the preoperative planning to maintain acute deformity correction and to prevent secondary dislocation of the distal fragment. A lateral blocking screw was inserted either if the nail was not in direct contact with the posterior cortex of the distal femur to prevent flexion deformity or for acute deformity correction in the sagittal plane. Intraoperative limb alignment was assessed by means of a sterile steel protractor. To assure correct guide wire and blocking screw positioning according to the planned correction the protractor was placed over the distal femur flush with the femoral condyles. Like this, limb alignment could be objectified by means of image intensifier intraoperatively. After blocking screw insertion, the intramedullary canal was opened with the entry reamer (Smith+Nephew) and then reamed 1.5–2.0 mm wider than the diameter of the planned nail using flexible reamers (Stryker, Kalamazoo, MI, USA), generally starting with a 7.5-mm flexible reamer. Corticotomy was performed with a multiple-drillhole technique with a 4.5-mm AO drill and subsequent chiseling with a 10-mm Lambotte osteotome. Iliotibial band release was not conducted in any of the patients.

#### Postoperative protocol

Distraction was started 7 days postoperatively, with 1 mm/day (2 x 0.5 mm). Patients were allowed partial weightbearing with 20 kg during distraction and physiotherapy was recommended twice a week. Lengthening was routinely conducted without external bracing. The mean hospitalization time for nail insertion was 9 days (standard deviation [SD] 3). Under distraction, patients underwent clinical and radiographic examinations every second week. After achieving the lengthening goal, a consolidation period of 6 weeks was initiated. If continuous cortical bone formation of at least 2/4 cortices of the femur was then confirmed on biplanar radiographs, full weightbearing was allowed, and patients were advised to refrain from sports for another 6 weeks. In total, 43/45 patients (96%) had implants removed after a median of 20 months (IQR 14–27) postoperatively and the median follow-up after implant removal was 16 months (IQR 6–35).

#### Limb-lengthening and limb-alignment parameters

Accuracy, precision, and reliability of the lengthening were calculated as previously described and analogously accuracy and precision of deformity correction were calculated using the mLDFA (Table 4) [6]. The level of osteotomy was measured on AP radiographs from the distal femoral joint line to the osteotomy on the lateral side of the nail. The distraction index (DIX) and consolidation index (CIX) were calculated as previously described [6]. Non-union was defined as lack of consolidation 6 months after end of distraction and treated by trauma nail stabilization (Trigen, Smith+Nephew, Memphis, TN, USA) with or without bone grafting. Delayed consolidation was retrospectively determined as a CIX greater than 2 SD of the mean CIX.

#### Complications

Treatment-related complications were reported descriptively and subclassified in complications not resulting in unplanned additional surgery and those resulting in unplanned additional surgery, and/or permanent sequelae [6,15].

#### Patient-reported outcome

The Limb Deformity-Scoliosis Research Society Score (LD-SRS-30), previously validated for the German language, was employed at final follow-up [16].

### Statistics

Normal distribution was assessed with the Shapiro–Wilk test. Descriptive statistics were conducted using mean with SD for normally distributed continuous variables, median with IQR for non-normally distributed continuous variables, and numbers with percentages for binary variables.

### Ethics, data sharing, funding, and disclosures

The study was approved by our institutional review board (registration number: 2019-368-f-S) and conducted according to the World Medical Association Declaration of Helsinki. The authors received no funding for this study. Data can be shared upon reasonable request via the corresponding authors. Complete disclosure of interest forms according to ICMJE are available on the article page, doi: 10.2340/17453674.2024.40947

## Results

In 9/45 patients (20%) treated between January and December 2013 the first generation (P1) and from 2014 onward in 36 patients (80%) the second generation PRECICE (P2) ILN was applied (NuVasive Specialized Orthopedics, Aliso Viejo, CA, USA) (Figure 1). The amount of additionally conducted surgeries, blood loss, operation and intraoperative fluoroscopy time were acquired from the surgical protocols (Tables 2 and 3).

**Table 2:** Perioperative parameters. Normally distributed values are presented with mean and standard deviation (SD) and non-normally distributed values with median and interquartile range (IQR)

Parameter	
At implantation	
Surgery time (SD), min.	144 (41)
Concomitant procedures, n/N (%)	24/45 (56)
Blood loss (IQR), mL ^a^	0 (0–50)
Fluoroscopy time (SD), min.	3.6 (1.7)
At removal	
Surgery time (IQR), min.	97 (55–161)
Concomitant procedures, n/N (%)	20/43 (47)
Blood loss (IQR), mL ^a^	0 (0–0)
Fluoroscopy time (IQR), min.	1.3 (0.5–2.2)

a Since the osteotomy is conducted with percutaneous technique the intramuscular hematoma was not included.

**Table 3:** Implant parameters

Length mm (n)	Diameter mm (n)	Stroke mm (n)	Implant type (n)
190 (1)	8.5 (9)	50 (23)	PRECICE P1 (9)
215 (16)	10.7 (28)	65 (5)	PRECICE P2 (36)
230 (3)	12.5 (8)	80 (17)	
245 (10)			
275 (9)			
305 (4)			
335 (2)			

**Table 4:** Parameters of distraction and deformity correction. For value presentation, see Table 2

Parameter	Entire cohort (n = 45)	Varus cohort (n = 16)	Valgus cohort (n = 29 ^a^)
Age (IQR), years	15 (13–19)	16 (13–28)	15 (14–17)
LLD (IQR), mm	41 (31–50)	42 (32–47)	40 (31–52)
MAD preoperative (IQR), mm	–15 (–26 to 23)	30 (23–38)	–25 (–29 to –15)
MAD postoperative (SD), mm	1 (11)	8 (8)	–3 (10)
mLDFA preoperative (IQR), °	82 (77–93)	96 (93–101)	79 (77–83)
mLDFA postoperative (SD), °	87 (5)	89 (5)	87 (5)
Planned angular correction (SD), °	–	11 (6)	10 (6)
Level of osteotomy (IQR), mm	81 (77–99)	82 (79–89)	81 (74–107)
Level of AP blocking screw (IQR), mm	74 (68–98)	75 (69–86)	71 (64–99)
Planned distraction (IQR), mm	47 (35–50)	49 (34–50)	45 (35–50)
Achieved distraction (IQR), mm	46 (29–49)	46 (31–50)	45 (29–49)
Difference planned vs achieved distraction (SD), mm	2 (3)	2 (3)	2 (3)
Planned distraction speed (SD), mm/day	1.0 (0.1)	0.9 (0.2)	1.0 (0)
Days under distraction (IQR)	51 (34–62)	57 (51–76)	48 (32–58)
Days under consolidation (IQR)	86 (50–124)	89 (87–154)	84 (47–108)
Distraction index (IQR), mm/day	0.9 (0.7–1.0)	0.9 (0.7–1.0)	0.9 (0.7–1.0)
Consolidation index (IQR), days/cm	29 (24–43)	35 (26–54)	29 (21–38)
Accuracy of distraction (IQR), % ^b^	96 (93–97)	93 (92–97)	96 (94–97)
Accuracy of angular correction (IQR), % ^c^	95 (89–98)	94 (89–96)	95 (89–98)
Precision of distraction, % ^d^	94	94	94
Precision of angular correction (%) ^e^	–	89	89
Reliability (%) f	96	94	94

a Including the 2 patients with flexion deformity of the distal femur.

b Accuracy of distraction = (100 – |(achieved distraction in mm – planned distraction in mm) / (planned distraction in mm) x 100|.

c Accuracy of angular correction = (100 – |(postoperative mLDFA – preoperative mLDFA – planned degree of correction) / (postoperative mLDFA) x 100|.

d Precision of distraction = 100 – relative SD of accuracy of distraction.

e Precision of angular correction = 100 – relative SD of angular correction.

f Reliability = 100 x number of lengthening nails in situ until osseous consolidation/total number of implanted lengthening nails.

### Lengthening and deformity correction

**Figure 4:**
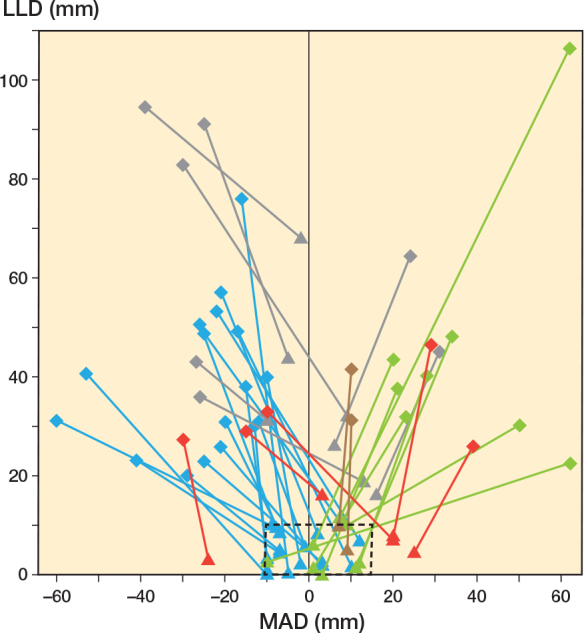
The spaghetti plot shows the change in leg length discrepancy (LLD) and mechanical axis deviation (MAD) as a result of the reconstruction from preoperative (diamond) to postoperative status (triangle) for the group with valgus deformity (n = 25), the group with varus deformity (n = 12) and the group with flexion deformity (n = 2). The cases in which an LLD measurement was not possible postoperatively, are not presented (n = 6). The normal range for LLD (≤ 10 mm) and MAD (–10 mm to 15 mm) is shown with a dashed outline. In the majority of corrections, this target corridor was achieved postoperatively (n = 27; valgus n = 17 [blue], varus n = 8 [green], flexion [no intended coronal angular correction] n = 2 [brown]). In all cases in which the residual LLD was > 10 mm multiple gradual lengthening procedures were planned from the outset (n = 7 [grey]). The cases in which no sufficient coronal angular correction could be achieved are shown in red (n = 5; undercorrection n = 4, overcorrection n = 1). All of these residual angular deformities were subsequently corrected sufficiently in further initially unplanned interventions. We caution the reader to correctly interpret this figure. A residual LLD does not mean that the planned distraction was not achieved. The difference between the planned and achieved distraction is depicted in Table 4. However, LLD and MAD were chosen because these parameters represent the primary treatment goals for the affected patients.”.

The median distraction was 46 mm (IQR 29–49), median DIX was 0.9 mm/day (IQR 0.7–1.0), and median CIX was 29 days/cm (IQR 24–43), respectively. The median preoperative MAD of the varus cohort was 30 mm (IQR 23–38) and –25 mm (IQR –29 to –15) in the valgus cohort and reduced to a mean of 8 mm (SD 8) and –3 (SD 10), respectively (Table 4). In total, 40/45 of patients (89%) achieved distraction with a difference of less than 1 cm from the initial plan and a postoperative MAD ranging from –10 mm to +15 mm. The inter-individual development of MAD and LLD correction for each patient is depicted in Figure 4.

### Clinical outcome and complications

Distraction was completed in 33/45 patients (73%) without the use of analgesics. Distraction-associated pain was sufficiently relieved by treatment with non-opioid analgesics in 10 and with a combination of non-opioid analgesics and oral opioids in 2 individuals. Distraction was completed at the initially planned distraction rate of 1 mm/day in 34/45 patients (76%) and was adjusted in 11 of the segments (accelerated: 1, decelerated: 5, temporarily paused: 6, temporary retracted: 3). The most common reason for decreasing the distraction rate was insufficient callus formation or extension deficit of the knee greater than 15°. Range of motion limitations of the knee during distraction were observed in 23/45 patients (51%) and resolved in 19 patients with physiotherapeutic treatment. Of the remaining 4 patients, 1 patient had isolated soft tissue contractures without subluxation of the knee and was treated by knee mobilization under general anesthesia and a thigh cast. The other 3 had knee subluxation and were treated surgically (Table 5).

**Table 5:** Analysis of major complications that led to unplanned additional surgeries (sorted by type of complication)

Patient no.	Type of major complication	Etiology	mLDFA (°)	LLD (mm)	Age (years)	Planned distraction/achieved (mm)	Type of additional surgery	Treatment period (nail)
1	Failing distraction due to implant malfunction	PT	48	102	15	47 /42	Re-osteotomy and exchange of lengthening nail	2013 (P1)
2	Dislocation of 1 distal locking bolt	Iatrogenic	41	75	16	45 /47	Repositioning of the same locking bolt	2013 (P1)
3	Non-union resulting in new varus and torsional deformity of the femur and implant failure	PT	40	67	30	50 /47	Implant removal, repositioning of blocking screws, re-osteotomy, and acute deformity correction with trauma nail insertion	2017 (P2)
4	Non-union	Post SCFE	45	79	25	45 /43	Nail exchange to trauma nail and autologous bone grafting from iliac crest	2018 (P2)
5	Non-union	PT	95	75	23	60 /60	Nail exchange to trauma nail and autologous internal bone grafting by intramedullary reaming	2019 (P2)
6	Non-union and insufficient deformity correction	PT	26	97	31	26 /27	Nail exchange to trauma nail, re-osteotomy, acute deformity correction, and autologous internal bone grafting by intramedullary reaming	2020 (P2)
7 ^a^	1. Insufficient correction of angular deformity due to malpositioning of blocking screw	CFD	43	84	15	40 /17	1. Removal of blocking screw and lengthening nail, acute angular correction with distal fragment control by temporary Steinman pin insertion, repositioning of 2 blocking screws, and reinsertion of lengthening nail	2016 (P2)
	2. Knee subluxation						2. Retraction of lengthening nail, closed reduction on traction table, and application of thigh cast for 6 weeks, lengthening with external fixator after 2 months	
8	Knee subluxation	CP, GMFCS I	31	85	15	32 /34	Lengthening of iliotibial band, closed reduction on traction table and application of thigh cast for 6 weeks	2015 (P2)
9	Knee subluxation and patellar dislocation	Tumor	43	107	9	60 /60	Extensive soft tissue reconstruction including open patellar reduction	2019 (P2)
10	Extension deficit of the knee after lengthening	Idiopathic	30	67	28	30 /23	Premature cessation of distraction due to extension deficit of the knee, which was subsequently corrected by a distal femoral extension osteotomy	2016 (P2)
11	New valgus deformity of distal femur after 46 mm of lengthening due to malpositioning of blocking screw	CFD	53	79	16	53 /51	Removal of blocking screw and repositioning of new blocking screw	2014 (P1)
12	Insufficient correction of angular deformity due to malpositioning of blocking screw	PI	47	95	13	47 /47	Insufficient blocking screw positioning	2015 (P2)
13	1. Septic arthritis of the knee after implant removal	PI	40	86	29	40 /39	1. Operative irrigation via mini-arthrotomy, intramedullary reaming of femur, local and systemic antibiotic treatment	2020 (P2)
	2. Persisting septic arthritis after first revision						2. Operative irrigation via arthroscopy and systemic antibiotic treatment	

Abbreviations: LLD = leg length discrepancy, mLDFA = mechanical lateral distal femoral angle, CFD = congenital femoral deficiency, PT = post-traumatic. NA = not available, CP = cerebral palsy, GMFCS = Gross Motor Function Classification System, CHD = congenital hip dislocation, SCFE = slipped capital femoral epiphysis, PI = postinfectious, P1 = first-generation Precice nail, P2 = second-generation Precice nail.

**Figure 5:**
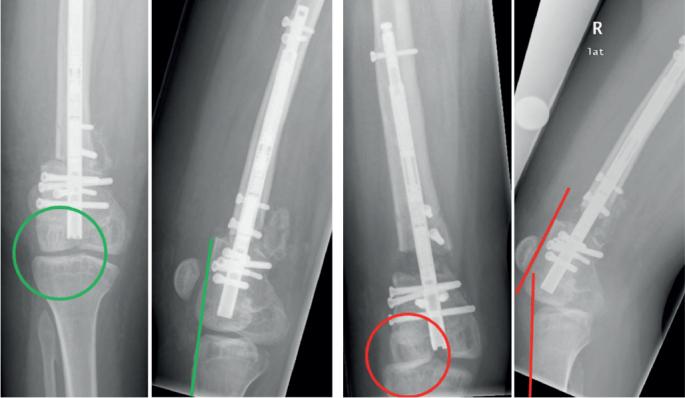
A 15-year-old female patient (patient 7 in Table 5) with congenital femoral shortening and distal femoral valgus deformity due to congenital femoral deficiency. Deformity reconstruction using a retrograde intramedullary lengthening nail (PRECICE, NuVasive) and multiple blocking bolts for acute angular correction with subsequent gradual lengthening by distraction osteogenesis (left panels). Immediately after nail implantation with angular deformity correction showing the typical flattening of the femoral notch and absence of the tibial intercondylar eminence as signs of cruciate ligament aplasia. The entire joint space is clearly visible in the anteroposterior (AP) view and the tangents to the anterior femoral and tibial edges coincide in the lateral view (right panels). After gradual lengthening of 15 mm a typical knee subluxation with extension deficit is seen. In AP view the lateral femoral condyles and tibial plateau overlap and the tangents to the femoral and tibial edges no longer coincide due to anterior translation of the femur in relation to the tibia.

a Radiographs of patient 7 are shown in Figure 5.

Unplanned additional surgeries were conducted in 13/45 patients (29%) but eventually the treatment goal was achieved in all these patients without permanent sequelae (Table 5). The most frequent reason for additional surgery was non-union in 4 patients, which was treated by nail exchange to a trauma nail with bone grafting. Delayed consolidation greater than 2 SD than the mean CIX occurred in 14 segments, of which 10 healed without additional surgery. The postoperative LD-SRS-30 score was available for 26/45 patients and resulted in a mean of 4.0 (SD 0.5).

## Discussion

The study aimed to analyze reliability, precision, and complications of acute angular deformity correction and gradual femoral lengthening via a retrograde ILN. Therefore, a homogeneous cohort in terms of the applied implant and ILN insertion technique was studied retrospectively. We found that accurate and precise acute angular deformity correction of the distal femur and subsequent gradual distraction osteogenesis can be conducted in 89% of patients. Accuracy and precision of lengthening ranged from 93–95% and accuracy and precision of angular correction from 89–92%.

With 45 patients this study comprises the largest cohort simultaneously treated by femoral distraction osteogenesis with magnetically controlled motorized ILN inserted via a retrograde approach and simultaneous acute angular deformity correction of the distal femur. Comparability to previous studies with an equivalent study design is limited, as Calder et al. (34 segments) [1], Iobst et al. (27 segments) [8], Teulières et al. (10 segments) [17], and Geiger et al. (41 segments) [7] do not provide these values. Accuracy and precision of this study fit in the range of studies that analyzed femoral distraction osteogenesis with antegrade nail insertion (range 86–97%) [6,18,19]. Regarding bone healing of patients treated with retrograde femoral ILN, a similar CIX (33 days/cm) was observed in our study to that of Calder et al., who found a CIX of 36 days/cm [1], Iobst et al. of 42 days/cm [8], and Geiger et al. of 28 days/cm (valgus cohort) and 35 days/cm (varus cohort) [7]. The average DIX of our study was 0.9 mm/day, which, in accordance with Karakoyun et al. [20], might indicate that the nail approach (antegrade/retrograde), the site of distraction (proximal/distal femur), and the amount of angular deformity correction do not lead to clinically relevant differences in terms of CIX and DIX. While these mean values regarding bone healing appear similar, we caution the reader that in this study insufficient callus formation requiring nail exchange and bone grafting was observed in 9% of patients. Calder et al. observed non-union in 10% of patients [1] and Geiger et al. reported delayed consolidation in 12% and nonunion in 7% [7]. This is more frequent than in cohorts treated with antegrade ILNs ranging from 0–4% [1,2,6]. One might hypothesize that an open wedge or translation at the osteotomy site might lead to disruption of the periosteum, which could negatively influence healing capacity. However, this assumption is not supported by available investigations on this topic [20,21]. Liska et al. found a higher non-union rate in lateral closing wedge osteotomies compared with opening wedge osteotomies of the distal femur [21]. This could indicate that translation of the distal femoral segment related to acute deformity correction with ILNs does not negatively influence consolidation. For retrograde ILN insertion, the osteotomy is generally placed at the junction between the metaphysis and the diaphysis, whereas for antegrade tibial ILN insertion metaphyseal osteotomies are usually possible, presumably allowing better healing conditions. This finding is supported by lower non-union rates reported by Vogt et al. and Wright et al. for tibial lengthening with ILNs [15,22]. Related to femoral lengthening with retrograde ILN, Geiger et al. reported 41% (17/41) [7], Iobst et al. 12% (3/25) [8] and Calder et al. 24% (8/34) [1] of unplanned additional operations. In accordance with 29% of unplanned additional surgeries in our cohort this indicates that in femoral lengthening with a retrograde ILN higher complication rates should be anticipated compared with femoral lengthening with antegrade ILNs (14-27%) [1,2,7,8]. The relatively high rates of unplanned additional surgery found in cohorts lengthened with retrograde femoral ILN could possibly be explained by the underlying etiologies, which, as in this study, are commonly post-traumatic, postinfectious, or, according to Geiger et al., congenital LLD with concomitant angular deformity [7]. However, based on a retrospective multicenter cohort study, Frost et al. found no increased relative risk of complications for bone lengthening and concomitant correction at the osteotomy site in 257 segments when adjusted for etiology and age [23]. One should especially be aware of the risk of delayed consolidation, soft-tissue contractures, or knee subluxation during and after treatment, as these represented the most severe complications in our cohort and in the studies of Geiger et al. [7] and Calder et al. [1]. Because of the results of this study, we avoid distal femoral lengthening via retrograde ILNs in patients with pre-existing knee instability to reduce the risk of iatrogenic knee subluxation. One might assume that lengthening of the iliotibial band at index surgery or additional bracing during distraction reduces this risk. However, there is no comparative study that might support this hypothesis.

As an alternative or in addition to blocking screws that control the distal fragment straight rigid reamers can be used instead of flexible reamers [2,14]. However, the use of rigid reamers might be riskier than flexible reamers due to the risk of accidental perforation of the cortex. Correct positioning of blocking screws is crucial for deformity correction. Instead of using the “reverse rule of thumb” [11] we applied a slightly different principle: “The blocking screw precedes the direction of fragment rotation for correction.” With this technique, 1 blocking screw in the distal fragment is usually sufficient because the medullary canal of the diaphyseal femur is narrow enough to stabilize the proximal fragment. Some authors routinely use the temporary placement of external fixators for intraoperative correction and stabilization of fragments after osteotomy before the ILN is implanted [1,7,8]. Our results show, in agreement with other studies [2,20], that without a fixator-assisted technique sufficient deformity correction with a retrograde ILN can be achieved. Furthermore, femoral lengthening using an antegrade ILN combined with distal growth-guiding correction of an angular deformity is an alternative option in skeletally immature patients and has shown good and reliable results [9]. One- or two-stage treatment including femoral lengthening with an antegrade ILN and subsequent or concomitant distal femoral osteotomy with plate fixation for angular deformity correction is also an option to discuss when counselling patients [10,11]. A review of literature by Wylie et al. of 372 isolated medial closing compared with lateral opening wedge osteotomies of the distal femur without lengthening procedures found 3.2% of nonunion and 3.8% delayed union rates, which is lower than in our cohort (9%) [24]. This indicates that acute deformity correction of the distal femur and simultaneous lengthening with ILNs might increase the risk of nonunion compared with isolated osteotomies. However, to date no study has reliably compared the potential risks, benefits, and patient-reported outcome of the aforementioned techniques.

### Limitations

During the study period different treatments for similar indications were conducted including antegrade ILNs, epiphysiodesis, external fixators, or isolated acute deformity correction of the distal femur. We tried to mitigate this bias by consistent indications for retrograde femoral ILN. Patients were treated by the same surgeons who assessed and interpreted the data, which is a source of assessment bias, and interpretation can be biased based on pathology. We caution the reader that the mean implant removal time in this cohort (22 months) is later than the official recommendation of the manufacturer, who suggests implant removal 1 year after implantation. In contrast to Krieg et al. who, in a retrospective long-term study 10 years after implantation of 13 retrograde femoral ILNs, found structural changes such as femoral and retropatellar cartilage damage and Hoffa fibrosis, but pain-free and non-restricted knee joints [25], no conclusion can be drawn regarding long-term effects on the knee joint related to retrograde ILN insertion from this study. The LD-SRS-30 was available only from 58% of patients and might not be representative of the entire cohort. The mean value of 4.0 is higher compared with patients with LLD (3.6) or LLD combined with angular deformities (3.5) [16,26] and fits in the range (4.0–4.3) of the few studies that assessed patient-reported outcomes after femoral and/or tibial distraction osteogenesis with ILNs [15,27,28].

### Conclusion

Acute angular deformity correction and subsequent gradual lengthening of the distal femur with a magnetically driven motorized ILN implanted via a retrograde approach is a reliable and accurate treatment for simultaneous correction of LLD and angular deformities, achieving the treatment goal in 89%. However, unplanned additional surgeries in 29% of patients should be anticipated. Lengthening-associated knee subluxation was the most severe complication, observed in 7% of patients.

## Notes

BV: Methodology, investigation, data curation, formal analysis, visualization, writing – original draft, writing – review & editing. CB: Conceptualization, methodology, investigation, data curation, formal analysis, GG: Resources, supervision, writing – review & editing. AL: Data curation, formal analysis, visualization, writing – review & editing. AR: Formal analysis, review & editing. CA: Formal analysis, data curation. ML: Investigation, data curation. GT: Visualization, data curation, review & editing HT: Resources, data curation. RR: Conceptualization, project administration, supervision, writing – review & editing. AF: Conceptualization, methodology, project administration, supervision, data curation, formal analysis, writing – original draft, writing – review & editing.

Handling co-editor: Ilkka Helenius

*Acta* thanks Franck Accadbled, Joachim Horn, and Søren Kold for help with peer review of this manuscript.
